# Effect of garlic on lipid profile and expression of LXR alpha in intestine and liver of hypercholesterolemic mice

**DOI:** 10.1186/2251-6581-13-20

**Published:** 2014-01-29

**Authors:** Abbas Mohammadi, Ebrahim Abbasi Oshaghi

**Affiliations:** 1 Department of Biochemistry, Afzalipour School of Medicine, Kerman University of Medical Sciences, Kerman, Iran; 2 Physiology Research Centre, Afzalipour School of Medicine, Kerman University of Medical Sciences, Kerman, Iran; 3 Department of Biochemistry, Medical School, Hamadan University of Medical Sciences, Hamadan, Iran; 4 Student Research Committee, Faculty of Medicine, Hamadan University of Medical Sciences, Hamadan, Iran

**Keywords:** Cholesterol, Garlic, LDL-C, LXR

## Abstract

**Background:**

Garlic is one of the medicinal plants which has showed beneficial effects on atherosclerosis risk factors. The liver X receptor α (LXRα) is an important regulator of cholesterol, triglyceride and glucose homeostasis that belongs to the nuclear receptor superfamily. In this study we investigated the effect of garlic on lipid profile, glucose as well as LXRα expression in intestine and liver of mice.

**Methods:**

Forty male N-Mary mice were randomly divided into 3 groups (n = 8): group1 received chow + 2% cholesterol + 0.5% cholic acid, group 2: chow + 4% (w/w) garlic extract + 2% cholesterol + 0.5% cholic acid, and group 3: chow only. After one month of treatment, mice were anesthetized, blood was collected from their heart, and the first 10 cm of the small intestine and liver were removed. Glucose was measured by a glucometer; other biochemical factors were measured by enzymatic methods. LXR expression was checked by RT-PCR and western blotting.

**Results:**

Compared with hypercholesterolemic mice, treatment with garlic extract significantly decreased total cholesterol, low-density lipoprotein cholesterol (LDL-C), triglycerides, very low density lipoprotein-cholesterol (VLDL-C), atherogenic index, alanine aminotranferease (ALT) and aspartate aminotransferase (AST) (all of them P < 0.05). Change in HDL-C levels was not significant in garlic-extract treated animals compared with hypercholesterolemic group. LXR protein and mRNA in the intestine were increased in garlic-extract treated group compared with chow group (P < 0.05), while in the liver, only mRNA of LXR was increased in hypercholesterolemic control mice (P < 0.05).

**Conclusions:**

The present study demonstrated that garlic extract reduced LXRα expression in the liver and increased its expression in the intestine. These effects probably have an important role in reducing serum triglyceride and cholesterol.

## Background

There are many risk factors for atherosclerosis including; high levels of total cholesterol, low-density lipoprotein cholesterol (LDL-C), triglycerides(TG) and a low level of high-density lipoprotein cholesterol (HLD-C) [1,2]. Statins which inhibit cholesterol synthesis are suggested as the first line in lipid lowering treatment. Many patients can not tolerate high doses of statins because of adverse effects, and the LDL-C levels are not adequately reduced, thus in these patients, alternative or combination drug treatment is a good option [3]. Garlic is one of the medicinal plants which in many animal and human studies have shown some beneficial effects on atherosclerosis risk factors. Many animal and human studies have shown that garlic extract is very appropriate for many diseases, because this medicine herb has a lot of useful effects, including; hypolipidemic and hypocholesterolemic effects, antimicrobial and antifungal activity, anticarcinogenic activities, antioxidant activity, anti-hypertensive activity, anti-diabetic, anti-hyperhomocysteinemia and antithrombotic effects [4].

The livers X receptors (LXRα (NR1H3) and LXRβ (NR1H2)) are important regulators of cholesterol, triglyceride and glucose homeostasis that belong to the nuclear receptor superfamily. LXRs with regulation of ATP-binding cassette transporter (ABC) genes have a critical role in the reverse cholesterol transport. It has been demonstrated that LXRα agonists effectively block intestinal cholesterol absorption. LXRα also plays a role in cholesterol storage and steroidogenesis pathways [5,6]. Wang Y, et al. have been shown that LXRα plays vital role in the controlling of cholesterol biosynthesis via directly silencing the expression of two main enzymes (squalene synthase and lanosterol 14α-demethylase (CYP51A1)) [7]. Zelcer N, et al. reported that LXRα regulate cholesterol metabolic by inducing of cholesterol exclusion and reverse cholesterol transport [8]. Resveratrol which has known as hypoglycemic and hypolipidemic agent inhibit lipogenesis in hepatocytes via LXRα-mediated mechanism [9]. Mohammadi, et al., showed that flaxseed significantly reduced lipid profile through activation of LXRα in the intestine and inhibition of LXRα in the liver [10]. Zhang Y, et al. suggested that activation of LXRs (except hepatic LXRs) have beneficial effect for cardiovascular disease treatment [11]. In this experiment we have investigated the useful effects of garlic on lipid profile and expression of LXR alpha, which is involved in lipid and carbohydrate metabolism, in the liver and intestine of mice fed a high-cholesterol diet.

## Methods

### Preparing animals

Forty male N-Mary mice were purchased from the animal house of Kerman University of Medical Sciences. Animals were maintained on a 12 h light/12 h dark cycle at 22 ± 1°C. After one week for acclimatization, they were randomly divided into 3 groups (n = 8): group1 (hypercholestrolemic group) received chow + 2% cholesterol + 0.5% cholic acid, group 2 (garlic treated group) received chow + 4% (w/w) garlic extract + 2% cholesterol + 0.5% cholic acid, and group (chow group) received chow only. Blood glucose, TG and cholesterol were measured before starting treatment. This study was approved by the Animal Research Ethics Committee of Kerman University of Medical Sciences, Kerman, Iran.

### Preparation of water garlic-extract

Garlic was purchased from the market. Cloves of garlic were washed with distilled water, crushed and dried. Twenty grams of shadow dried crusehd garlic was pulverized and macerated in 200 ml of distilled water at room temperature for 24 h. After filtration, the extract was dried at 40°C in an incubator for 24 h. The extract was kept in dark vials at -20°C.

### Preparation of garlic-extract supplemented chow

Garlic extract (4 g per 100 g of chow) was dissolved in normal saline and mixed with chow. The same amount of normal saline was added to the diets of hypercholesterolemic and chow groups.

Animal treatments and preparation of serum, liver and intestine tissues.

Depending on the group that they were assigned to, animals had free access to garlic-extract supplemented, cholesterol supplemented, or normal chow. They were monitored daily and body weight was recorded every two days [12]. After one month of treatment, food was removed 12 hrs before the experiments. Mice were anesthetized with diethyl ether, and blood was collected from their hearts. Duodenum and liver were removed, washed with phosphate buffered saline (PBS), and stored in – 70°C till use. Liver was weighed before freezing.

### Biochemical factors

Serum was obtained by centrifugation of blood samples at 3000 *g* for 10 minutes and stored at -20°C until assay. HDL-C, total cholesterol, triglycerides, alanine aminotransferase (ALT) and aspartate aminotransferase (AST) activities were analyzed using commercial kits (Pars Azmon, Tehran, Iran). Fasting blood glucose was measured by a glucometer (Roche). The LDL-C and VLDL-C levels were calculated using Friedwald equation. Atherogenic index (AI) was calculated using the following equation [12-17]:

$$\mathrm{Atherogenic}\;\mathrm{index}=\frac{\mathrm{total}\;\mathrm{cholesterol}-\left(\mathrm{HDL}-C\right)}{\mathrm{HDL}-C}$$

### Reverse transcriptase PCR (RT-PCR)

Frozen liver and intestine were used to extract RNA. Total RNA from liver and intestine were extracted using Accuzol Reagent (Bioneer, Korea) according to the manufacturers’ protocol.

Synthesis of cDNA was performed according to the manufacturer’s protocol (Fermentas, Lithuania). briefly, 1 μl forward primer, 1 μl reverse primer, 12 μl PCR Master Mix, 2 μl cDNA and 9 μl deionized water were added into a sterile pipe on ice. For RT- PCR reaction, thirty five cycles of PCR amplification were performed with denaturation at 95°C for 30 s, annealing at 63°C for 30 s, and extension at 72°C for 30 s using a PCR machine. All PCR reactions were completed with a single extension cycle at 72°C for 5 minutes. The products were electrophoresed on a 2.5% agarose gel and visualized by staining with ethidium bromide. The following primers were used in this study. Mouse LXR alpha primer; F: 5′-GCG TCC ATT CAG AGC AAG TGT-3′ and R: 5′-TCA CTC GTG GAC ATC CCA GAT-3′. Mouse Beta actin Primer; F: 5′-TGG AAT CCT GTG GCA TCC ATG AAA C-3′ and R: 5′-TAA AAC GCA GCT CAG TAA CAG TCC G-3′ [13,15,18,19].

### Western blotting

For protein analysis 50 mg of liver or intestine were homogenized in 700 μl of RIPA buffer containing 1% protein inhibitor cocktail (Santa Cruz, USA) and 1 μM PMSF, then centrifuged (14000 rpm at 4°C for 15 minutes). Protein concentration of the samples was measured in a Nano Drop spectrophotometer and 120 μg of protein was loaded on a 12.5% SDS-PAGE gel. After electrophoresis, proteins were transferred to a PVDF (Roche Applied Science) membrane. The Membrane was blocked with 3% skim milk in Tris-buffered saline containing Tween-20 (TBS-T) (Roche Applied Science) for 2 hrs at room temperature. The membrane was washed (three times, 15 min each) in TBS-T and then incubated with primary antibody for 1.5 hrs with anti rabbit polyclonal antibody LXR (1:300 dilution, Novus Biological (NB400-157)). After 3 washes in TBS-T, the blots were incubated with horseradish peroxidase-conjugated anti-rabbit IgG (1:10,000 dilutions, Roche Applied Science) at room temperature for 1.5 hrs. The membranes were washed and exposed to ECL western blotting detection reagents (Roche Applied Science) and then exposed to films for 30s to 1 minute. Films were developed, scanned, and band densities were measured with Lab Work analyzing software (UVP, UK). Data are expressed as the percent ratio of the protein bands to *β*- Actin band [10,13,16].

### Statistical analysis

All results are presented as mean ± S.E.M. Statistical analysis of the results was done with one-way analysis of variance with ANOVA (Tukey). SPSS 14.0 for windows (SPSS Inc., Chicago, USA) was used for data analysis and different were considered significant at P < 0.05.

## Results

### Biochemical factors

Table 1 shows body weight, FBS, TC, LDL-C, HDL-C, TG and VLDL-C, in mice fed a high cholesterol diet, garlic extract and chow. There was a non significant reduction in blood glucose in garlic extract- treated group in comparison with hypercholestrolemic group. Serum TC, LDL-C, TG and VLDL-C significantly decreased in garlic extract-treated group. Change in serum HDL-C levels was not significant in garlic extract-treated group compared with hypercholestrolemic group, but markedly increased in comparison with chow. Table 2 shows AST and ALT in mice fed a high cholesterol diet, garlic extract and chow.

**Table 1:** Comparison of biochemical factors between different treatment groups

**Biochemical factors**	**Hypercholesterolemic**	**Garlic**	**Chow**
Body weight (g)	37.5 ± 1.5^f^	35.7 ± 0.8	30.7 ± 0.7
FBS (mg/dl)	160.1 ± 5.6	152.7 ± 4.6	138.1 ± 9.7
TC(mg/dl)	230.1 ± 4.5^f^	191.4 ± 5.4^a^	129 ± 13.4
TG (mg/dl)	160.5 ± 7.5^d^	137.4 ± 4.5^a^	133.5 ± 4.0
VLDL-C (mg/dl)	32.1 ± 1.5^d^	27.5 ± 0.9^a^	26.7 ± 0.8
HDL-C (mg/dl)	90.3 ± 8.1	106.2 ± 3.8^c^	87.4 ± 9.7
LDL-C (mg/dl)	98.8 ± 7.8^f^	57.6 ± 7.4^a^	24.9 ± 5.5
AI	1.6 ± 0.08^f^	0.8 ± 0.02^b^	0.48 ± 0.01

FBS: Fasting blood sugar, TC: Total cholesterol, TG: Triglyceride, HDL-C: High-density lipoprotein cholesterol, LDL-C: Low-density lipoprotein cholesterol, VLDL-C: Very low-density lipoprotein cholesterol, AI: atherogenic index. Data represent as mean ± SEM (n = 8).

^a^p < 0.05, ^b^p < 0.01 and ^c^p < 0.001 garlic group compared with hypercholestrolemic. ^d^p < 0.01 and ^f^p < 0.001 hypercholestrolemic group compared with chow.

**Table 2:** Comparison of liver enzyme between different treatment groups

**Biochemical factors**	**Control**	**Garlic**	**Chow**
AST (IU/L)	59.5 ± 4.5	43.7 ± 4.1^*^	42.5 ± 5.2
ALT (IU/L)	61.7 ± 4.6	42.1 ± 3.7^*^	41.5 ± 4.0

AST: Aspartate aminotransferase, ALT: Alanine aminotransferase. *p < 0.05 garlic compared with control (hypercjolesterolemic) (n = 8).

### RT-PCR analysis

**Figure 1:**
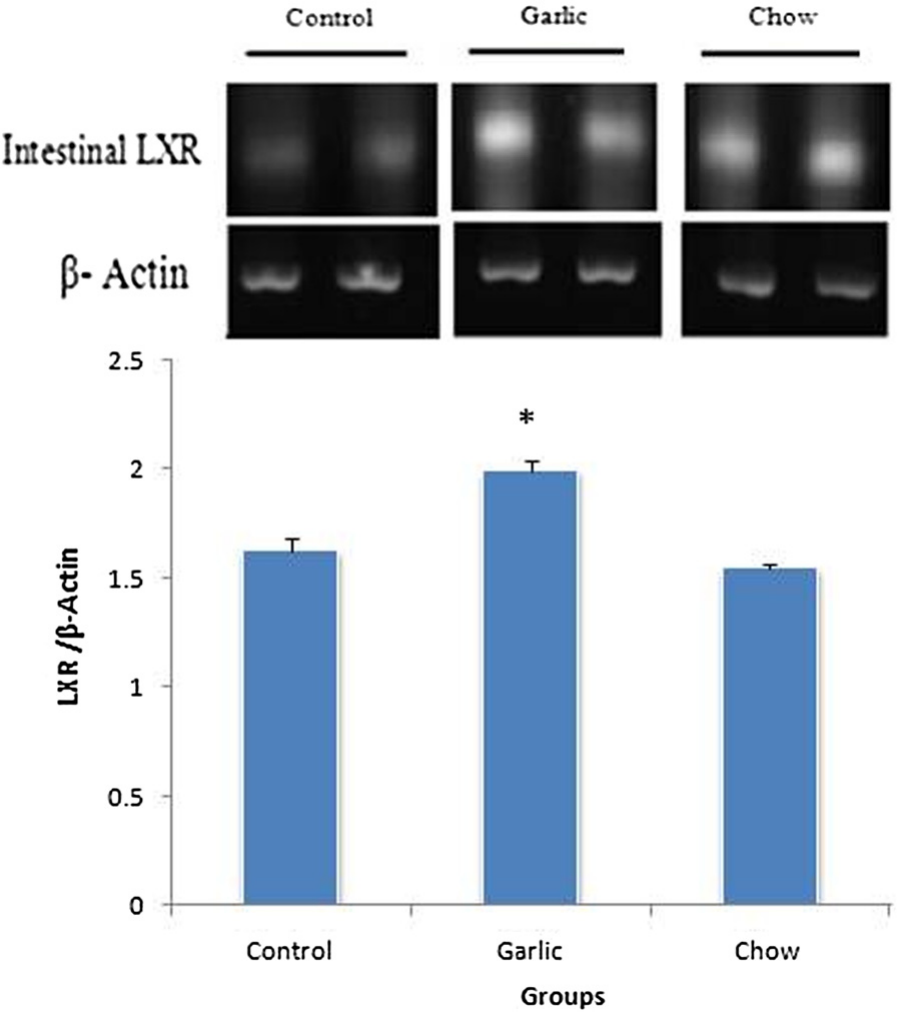
**Levels of LXR mRNA in the intestine of control (hypercholesterolemic), garlic and chow groups (n = 6–8).** Level of LXR mRNA was increased in garlic group compared with hypercholesterolemic group (*p < 0.05). Data are presented as mean ± SEM.

**Figure 2:**
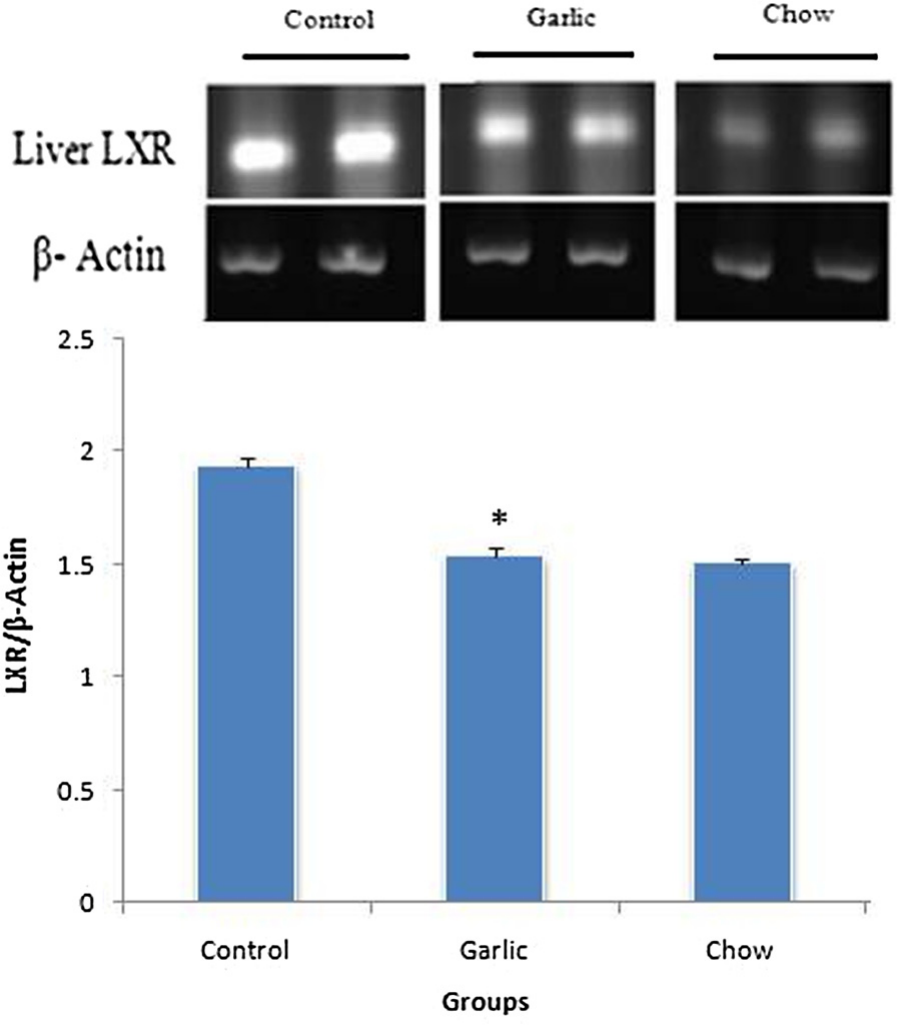
**Levels of LXR mRNA in the liver of control (hypercholesterolemic), garlic and chow groups (n = 6–8).** LXR mRNA level was reduced in garlic group compared with hypercholesterolemic group (*p < 0.05). Data are presented as mean ± SEM.

PCR products of LXR showed an expected band of 88 bp respectively. Intestinal and liver LXR mRNA markedly increased in garlic extract treated group compared with others groups (P < 0.05) (Figures 1 and 2).

### Immunoblot analysis

**Figure 3:**
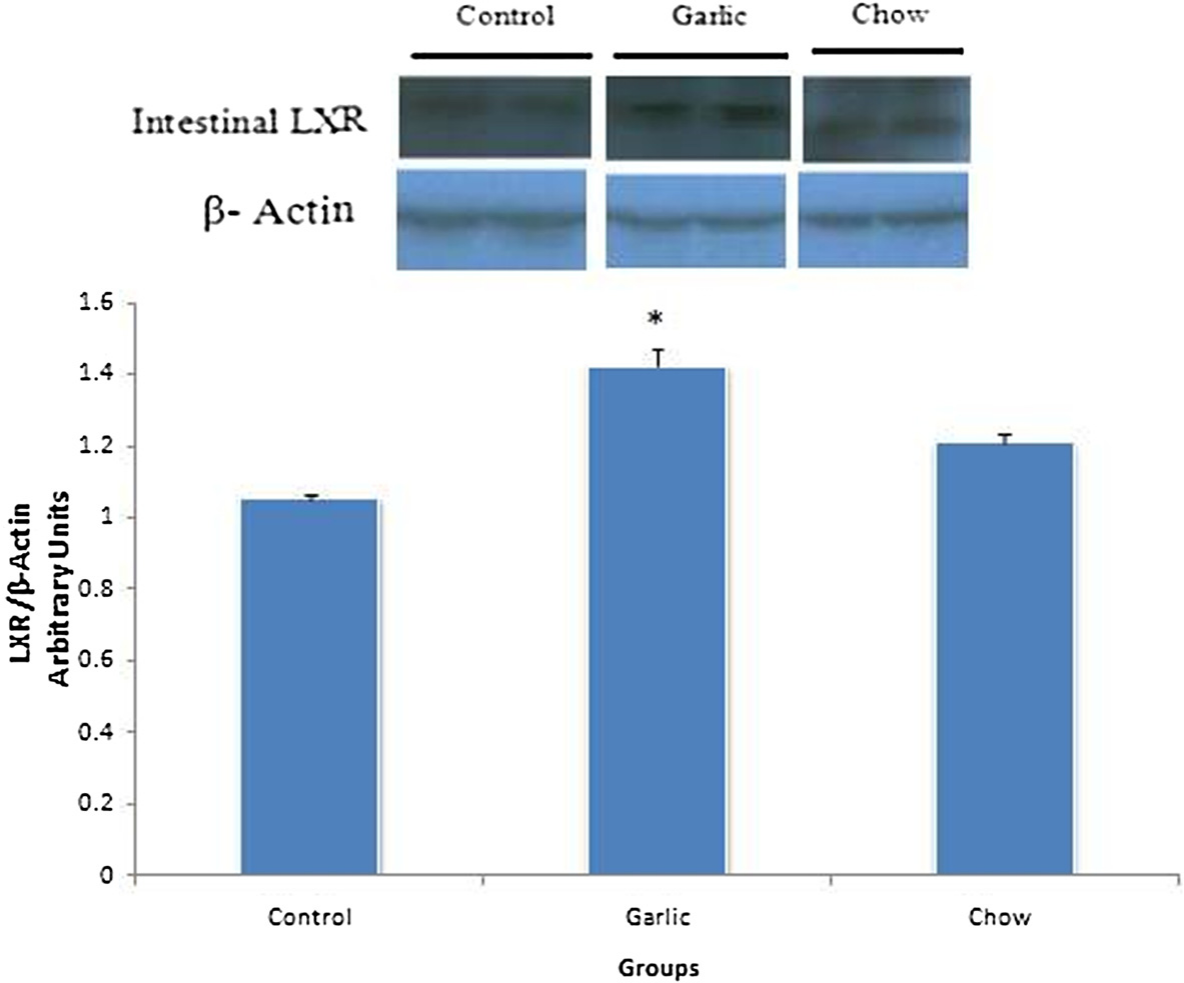
**Levels of LXR protein in the intestine of control (hypercholesterolemic), garlic and chow groups (n = 6–8).** LXR expression was increased in garlic group compared with hyppercholesterolemic group (*p < 0.05). Data are presented as mean ± SEM.

**Figure 4:**
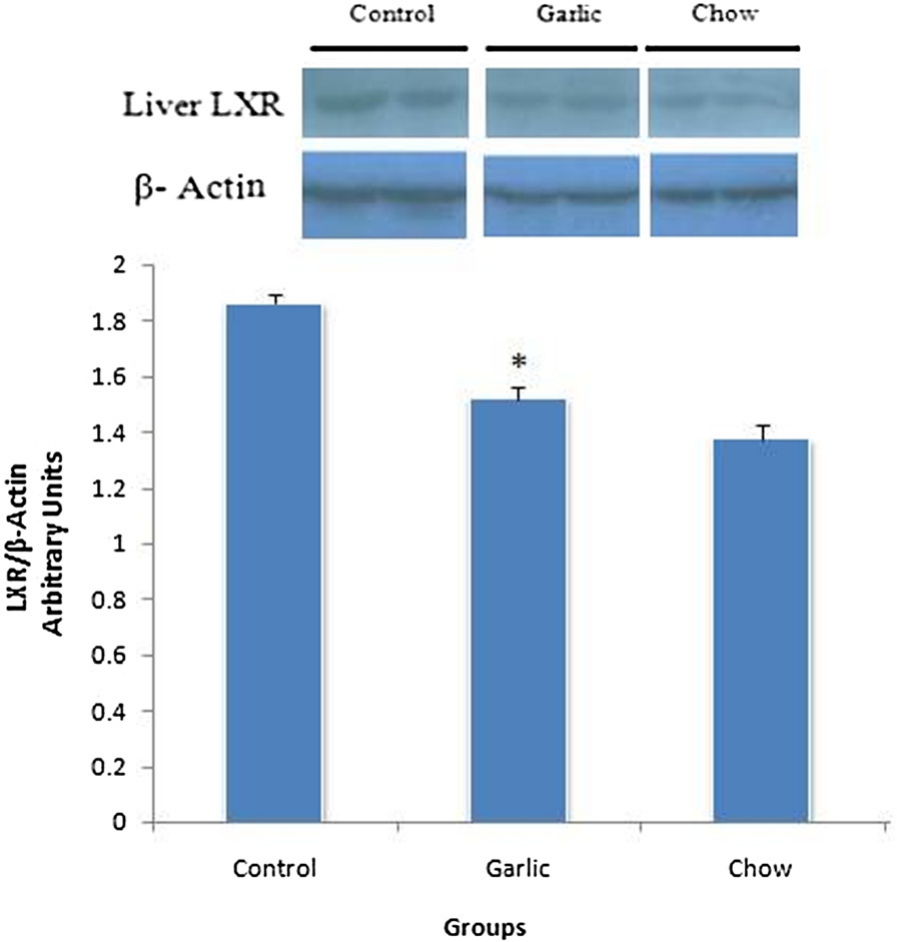
**Levels of LXR protein in the liver cells of control (hypercholesterolemic), garlic and chow groups (n = 6–8).** LXR protein expression was reduced in garlic group compared with hypercholesterolemic group (*p < 0.05). Data are presented as mean ± SEM.

Immunoblot analysis of the intestine and liver protein probed with anti LXR revealed bands with expected size of 64 KDa. Intestine LXR markedly increased in garlic extract-treated group hypercholesterolemic control (P < 0.05), while the LXR in liver was significantly reduced in garlic extract treated group (Figures 3 and 4).

## Discussion

Many human and animal studies, mostly short term, have investigated the lipid lowering effects of garlic. Cheng et al. showed that the maximum tolerable level of water-extracted garlic in diet was 10% in mice [20]. In the present study, we used a diet containing 4% w/w garlic extract in order to be well tolerated by the animals. Adding the garlic extract to the food did not change the food intake and body weight. Results from Yu-Yan Yeh et al. study suggest that the water-soluble sulfur compounds in garlic inhibit cholesterol synthesis, whereas the inhibition by lipid-soluble extracts results from the strong cytotoxic effect of this extract [21]. They also showed that water-extract inhibited cholesterol synthesis more efficiently than methanol-extractable fraction and petroleum ether-extractable fraction [21]. Therefore, we used water extract in this experiment.

In our study garlic extract significantly reduced total serum cholesterol (16.9%), LDL-C (30%), triglycerides (14.3%), VLDL-C (15.5%), compared with hypercholesterolemic control (cholesterol 2% and 0.5% cholic acid). Elmahdi B et al. reported that adding 8% raw garlic along with 2% cholesterol to rat diet, decreased plasma total cholesterol and LDL-C and increased HDL-C [22]. LDL-C reduction by garlic extract may be due to decreases of hepatic 3-hydroxy-3-methylglutaryl-CoA reductase, cholesterol 7α-hydroxylase, pentose-phosphate pathway activities [23], cholesteryl ester transfer protein activity [24], microsomal triglyceride transfer protein [25], increased bile acid excretion [26] and inhibition of hepatic fatty acid synthesis [27]. Aouadi et al. showed that adding 10% fresh crushed garlic and 2% cholesterol to diet led to significant reduction in LDL-C levels, and increased HDL-C levels in rats [28]. In our study, HDL-C level was significantly increased in garlic extract group compared to chow group. The results of our study also show that fasting blood glucose levels decreased in garlic group compared with hypercholesterolemic group but, it was not significant. Aouadi R and Ali M, [28,29] reported that garlic has no effect on blood glucose. Similar to Ali M et al. [29] in our study there was no change in body weight in the groups that consumed a high cholesterol diet with garlic. In the present study, we found that garlic extract significantly reduced plasma levels of ALT and AST. Furthermore, the observed decrease in serum AST and ALT activities by garlic extract also reflects decreased high cholesterol diet induced hepatocyte injury in mice.

Overall activation of LXR with synthetic agonists has several effects; hypertriglyceridemia, high HDL levels, hepatic steatosis, increased excretion of biliary cholesterol, reduced absorption of intestinal cholesterol and increased fecal neutral sterol excretion. LXRα activation in intestine leads to reduction of intestinal cholesterol absorption via increased ABCG5 and ABCG8 expression and reduction of intestinal NPC1L1 expression [6]. Furthermore, activation of LXR leads to markedly increased levels of ABCA1 mRNA and consequently increased HDL levels. Not only LXR agonist, but also high cholesterol, up-regulated expression of these genes via LXR, so it has been proposed that biological role of LXR is to recognize high concentrations of intracellular cholesterol and to prevent of cholesterol accumulation in the cell [5,30]. In this study garlic markedly increased LXR mRNA and protein in the intestine. We showed that garlic significantly reduced intestinal NPC1L1 expression and increased ABCG5 and ABCG8 expression in the intestine (unpublished). Therefore, we can conclude that decrease of blood cholesterol and LDL-C levels maybe is due to upregulation of intestinal NPC1L1, ABCG5 and Abcg8 expression via LXRα [5].

In rodents, LXRα increases catabolism of hepatic cholesterol and formation of bile acids by provoke of cholesterol 7α-hydroxylase, the rate-limiting enzyme which convert cholesterol to bile acids. activation of LXRα also display undesirable effects that cause steatosis and hypertriglyceridemia through stimulation of de novo hepatic lipogenesis via activating the transcription of lipogenic genes. A previous experiments showed that treatment with an LXRα agonist provoke the expression of lipogenesis genes [5,31].

In addition to cholesterol homeostasis LXRs have also been revealed to control biosynthesis of hepatic fatty acid. This procedure is governed by sterol regulatory element binding protein-1c (SREBP-1c) that regulates all the genes involved in this pathway, including acetyl-CoA carboxylase (ACC), fatty acid synthase (FAS) and stearoyl-CoA desaturase (SCD). Administration of LXR agonist, T0901317, raises hepatic expression of SREBP-1c, FAS, ACC, and SCD in wild type mice. T0901317-induced activation of lipogenesis leads to huge hepatic triglycerides accumulation and eventually to liver steatosis, liver dysfunction, and hypertriglyceridemia [5,6]. In the present study, we observed that garlic extract significantly reduced liver LXR α expression. Therefore, with reduction of liver LXR expression by garlic extract, we propose one of the mechanisms which garlic leads to reduction of blood triglyceride levels is this route.

## Conclusion

In summary, the present study demonstrated that garlic extract antagonized LXRα expression in the liver while; provoke LXRα expression in the intestine. Reverse expression of LXRα in these tissues maybe have important role in reduction of triglyceride and cholesterol by garlic. Combining this new result with prior information collected about the LXR importance and garlic useful properties it is probable to make this suggest that garlic could use as a potential agent to treatment of diabetes and CVD. Future studies need to investigate and approve garlic as a potential therapeutic target and LXR agonist.

## Competing interests

The authors declare that they have no competing interests.

## Authors’ contributions

AM designed the study and manuscript. ABO carried out the molecular genetic studies, participated in the gene expression and drafted the manuscript. Both authors read and approved the final manuscript.
